# Psychometric Analysis of the Quarantine Coping Strategies Scale (Q-COPE) in the Spanish Language

**DOI:** 10.3390/ijerph192214847

**Published:** 2022-11-11

**Authors:** Denis Frank Cunza-Aranzábal, Wilter C. Morales-García, Jacksaint Saintila, Salomón Huancahuire-Vega, Percy G. Ruiz Mamani

**Affiliations:** 1 Unidad de Posgrado de Ciencias Humanas y Educación, Universidad Peruana Unión (UPeU), Lima 15464, Peru; 2 Unidad de Posgrado de Salud Pública, Universidad Peruana Unión (UPeU), Lima 15464, Peru; 3 Escuela de Medicina Humana, Universidad Señor de Sipán, Chiclayo 14000, Peru; 4 Escuela de Medicina, Universidad Peruana Unión (UPeU), Lima 15464, Peru; 5 Escuela Profesional de Enfermería, Facultad de Ciencias de la Salud, Universidad Privada San Juan Bautista, Lima 15067, Peru

**Keywords:** coping, quarantine, pandemic, COVID-19, exploratory factor analysis, confirmatory factor analysis, validity, reliability

## Abstract

The possibility of facing an epidemic or pandemic resulting in mandatory isolation or quarantine has become a relevant construct for comparing and evaluating coping strategies under such conditions. The objective of this research was to develop and analyze the psychometric properties of a scale to assess quarantine coping strategies (Q-COPE). This was an instrumental study and 1110 Peruvian adults (M = 26.9 years; SD = 9.77) participated in the context of social isolation. For the construction of the scale, qualitative and quantitative procedures were followed. The internal structure was evaluated by exploratory factor analysis (EFA) and confirmatory factor analysis (CFA). The content analysis by expert judges supports the representativeness of the items related to the construct. EFA and CFA allowed the establishment of five factors: Emotional regulation, Information, Accommodation, Social support, and Altruism. The first-order model presents adequate goodness-of-fit indices: χ^2^ = 489.048, df = 220, χ^2^/df = 2.223, SRMR = 0.025, CFI = 0.969, TLI = 0.965, RMSEA = 0.047. Likewise, the second order model presented similar values: χ^2^ = 499.674, df = 225, χ^2^/df = 2.221, SRMR = 0.026, CFI = 0.969, TLI = 0.965, RMSEA = 0.047. The 23-item version was consistent with the proposed theory, obtained adequate fit indices and acceptable factor loadings (>0.70), and presented good internal consistency indexes evaluated by Cronbach’s α, ordinal α, omega (ω), and H coefficient. It is concluded that the Q-COPE scale presents good psychometric properties that justify its use in an adult population and allows the assessment of the coping strategies that people use in the face of a quarantine situation.

## 1. Introduction

The COVID-19 pandemic has impacted all aspects of individuals’ lives [1]. Restriction policies due to the virus have impeded economic and social activities. Meanwhile, quarantine or related events increased psychological distress and have wreaked havoc on the mental health of the population [2]. Thus, confinement as a containment strategy in the spread of the disease, despite receiving positive evaluations, had significant psychological effects [3,4,5,6]. Reduced social interaction and little objective information about the disease worsened people’s psychological health [7]. Various researches indicated that prolonged duration of confinement, fear of infection, financial loss, and inadequate information have led to seven times more increased depressive factors in 2020 (25%) compared to 2017 (3.44%) [8,9]. Psychological problems [10] also increased, such as panic disorder, anxiety [11], as well as increased frustration and uncertainty [12]. Similarly, social factors, such as low educational level, middle age, people living alone and with comorbidities or any health problems, led to reports of a greater deterioration in mental health [13,14,15,16]. In addition, psychological distress and psychological disorders were conveyed to children whose parents evidenced greater psychological impact due to increased financial worries, job loss, and lack of resources for daily needs such as food or water [17,18,19,20]. In light of this, studies indicate that positive mental health, resilience, and other strategies are often protective adaptive coping factors in situations of social isolation [21,22,23]. However, the lack of psychological resources limited healthy strategies in the face of the prevalence of psychological distress and psychiatric morbidity during the pandemic [24,25].

### Coping Quarantine

It is possible to confront and reduce the psychological impact of quarantine on two fronts: First, agents external to the individual, such as the government or health organizations. Actions taken by agents external to the individual include: reduction of quarantine time, which helps reduce post-traumatic stress, anger, and avoidance behaviors [26,27,28], provision of supplies [19,29], and information about the disease [30,31,32,33,34]. Second, the ways of thinking and acting that each individual possesses [19]. Thus, individuals can manage internal and external demands as a process of adaptation to perceived demands [35]. Thus, coping strategies include various consciously performed actions or thought processes used to directly confront unpleasant or stressful situations, modifying the response to a given situation [36]. Coping generally manifests itself in three forms: (a) rational, problem-centered coping that modifies the relationship of the environment with various actions; (b) emotional, emotion-centered coping that modifies the interpretation of the environment; and (c) avoidance, in which social distraction is sought [37]. These coping strategies can be of change and acceptance and can be developed over the long term through subjective experience and personal qualities.

Likewise, the three manifestations of coping in conditions of isolation can be distributed into categories: (a) Emotional regulation, which refers to expressing constructive emotions in the right place at the right time and is related to self-stimulation, emotional control, emotional expression, and relaxation [38] that will help reduce frustration by making sense of the quarantine [39], i.e., having an adaptive perception of control reinforcing the protective effect of positive mental health in coping with stress [21]; (b) the search for information, in relation to the knowledge of the situation, as well as intervention and mediation strategies [38] seeking access to timely and reliable information [14,40]. Thus, access to specific, current, and reliable health care information during quarantine is associated with less psychological impact [14,40]; (c) social support indicates seeking support from friends, family, and professionals through electronic means to obtain help or emotional support. Social support helps to cope with and reduce symptoms of anxiety and fear of the disease [41,42]; (d) altruism, focusing on the well-being of others who seek to conform to limitations in a sense of altruism [38] by following quarantine guidelines [19,43,44]. During the quarantine period, it is possible to engage in new activities, find moments of peace and calm, improve family ties, as well as enter into deep and meaningful contact with nature [39]; (e) accommodation, which seeks to adjust personal preferences to the constraints of the situation during the pandemic, is to accommodate limited access to supplies [38] provided by the state or some other means of production or provisioning [12,39,45,46]. While the distribution of supplies is more of a governmental responsibility, it is having the certainty of access to them [45,46]. Therefore, knowledge and understanding of coping strategies can reduce the psychological impact of quarantine and generate evidence for additional interventions in similar emergency situations.

Given this scenario, valid and reliable measures are needed to assess coping strategies in the face of quarantine. However, no instruments have been developed or validated for the general population to assess these coping strategies [47]. In the literature, instruments have been described that consider up to 17 coping strategies in different situations [48] Whereas, in specific pandemic quarantine situations, the most commonly used coping strategies include: taking preventive measures, actively learning about the disease, adjusting attitude toward the disease to cope positively, accessing supplies, and staying in communication with family and friends [49,50]. While prayer has been the most used religious coping mechanism, in a smaller percentage, having a daily routine and the use of music were also considered adequate coping strategies [51]. Likewise, the strategy most used by people without chronic diseases has been informational support to cope with the disease that motivated the quarantine [52].

The literature mentions that the potential risk of facing possible future isolation conditions requires planning and development of strategies to deal with them, with the purpose of mitigating their effects on people’s mental health [53]. Based on the above, specific instruments with evidence of validity and reliability are needed to measure coping strategies in the face of quarantine based on individual ways of thinking and acting that allow a person to adaptively manage their emotions, promote health and reduce the harmful psychological effects of a situation of isolation due to an illness. Therefore, the proposed construct includes five main themes: (1) ways of thinking and acting conducive to adaptive adjustment, (2) the management of information about the disease for which quarantine is performed, (3) social support through communication with people who provide it, (4) the sense of altruism, and (5) the provision of supplies.

In this sense, the general objective of this study is to design and evaluate an instrument to measure coping strategies in the face of quarantine. It also has specific objectives, such as: to assess the content validity of the Q-COPE items and to examine their internal structure, as well as to assess convergent and discriminant validity.

## 2. Materials and Methods

The study design is instrumental [54] because it involves the analysis of the validity and reliability of a test designed on the basis of a construct elaborated from a review of the available scientific literature.

### 2.1. Participants

This study considered the participation of 1110 Peruvian nationals, 552 (49.7%) males and 558 (50.3%) females, aged between 18 and 70 years (Mean = 26.9; SD = 9.77). According to the academic level, 483 (43.5%) were university students, 201 (18.1%) had a professional degree, 154 (13.9%) had a high school level of education, 114 (10.3%) were technicians, 87 (7.8%) had a bachelor’s degree, 33 (3.0%) had a master’s degree, 24 had incomplete university studies, 5 (0.5%) had a doctoral degree, 4 (0.4%) had only an elementary level, and 1 (0.1%) had incomplete high school.

### 2.2. Procedure

This research was carried out during the mandatory social isolation established by the Peruvian government. The survey was administered using a Google forms form (available from 17 July 2020 to 29 August 2020) sent through social networks (Facebook and WhatsApp). The first section of the form contained the informed consent, and the confidentiality of the information was guaranteed. It was also indicated that participants could withdraw from the study at any time. The study protocol was reviewed by a private Peruvian university (Approval number: CE-EPG-000092) and following the guidelines established in the Declaration of Helsinki.

### 2.3. Instrument

The Quarantine Coping Scale (Q-COPE) was initially developed taking into account the literature review, the classification of the three forms of rational [55], emotional, and avoidance coping [37] and five categories: emotional regulation, information, social support, altruism, and accommodation. A review of 24 empirical studies of qualitative, quantitative and mixed designs, conducted in several countries, including China, South Korea, Sweden, Australia, Sierra Leone, Senegal, Hong Kong, Taiwan, Liberia, Canada, and United States, in situations of quarantine due to various diseases, such as SARS, H1N1, Ebola, MERS, and equine influenza, was considered [19].

A total of 43 items were elaborated and analyzed by 2 psychologists who considered the categories of emotional regulation [38], supported by other studies that address the advantages of attitudes that reduce frustration [39], search for reliable information [14,40], accommodations in accessing necessary supplies [45,46], social support [41,42] and altruism [19,43,44], establishing from these sources a table of specifications [56] with 5 contents: Emotional regulation (15 items), information (9 items), accommodation (9 items), social support (6 items), and altruism (4 items) with 3 types of manifestations: rational (13 items), emotional (20 items) and avoidance (10 items), giving emphasis to the most relevant contents and manifestations, which had a greater number of items, prioritizing clarity and simplicity, avoiding technicalities, negations and excessive prolixity, also avoiding ambiguity [57].

The instrument is intended to assess coping strategies in conditions of enforced social isolation from the individual perspective. Items have 5 Likert-type response options: strongly disagree = 1, disagree = 2, neither agree nor disagree = 3, agree = 4, and strongly agree = 5; except for the reverse-rated items: 2–5, 8, 12–14, 16, 20, 30, 31, and 43 of the initial version.

### 2.4. Validation by Judges

To determine content validity, 4 psychologists with experience in academic research and instrument validation were asked to verify on a scale of 0 to 6 the overall fulfillment of the following criteria: item comprehension, appropriateness of the words to the context, and relationship of the item with its corresponding dimension and with the construct. Additionally, the following data were obtained from the evaluation of expert judges on the four criteria considered together: item comprehension, appropriateness of the words to the context, and the relationship of the item with its corresponding dimension and with the construct. Judges rated the criteria on a scale of 0 to 6 and their scores were quantified using the Aiken V validity index [58,59]. Considering the influence of sampling error and the need for an indicator for the practical usefulness of the results, 95% confidence intervals (95% CI) were calculated with the critical value of V = 0.50 as a criterion for review or rejection of each item [60].

### 2.5. Statistical Analysis

Prior to the descriptive analysis of the data, the total number of participants was divided into two samples of 555 participants each, with the purpose of using the first one for the exploratory factor analysis (EFA) and the second one for the confirmatory factor analysis (CFA) of the items. The division of the sample into two parts was done for cross-validation, as the EFA sample was used to develop the model and the CFA sample was used to test the model [36]. The descriptive analysis of the items was performed considering skewness and kurtosis, in which values between −2 and +2 indicated a distribution approaching normal [61,62]. Likewise, Mardia’s coefficient was used for multivariate normality analysis [63,64]. The adequacy of the sample for the EFA was assessed through the KMO index > 0.9, which would indicate the best fit [65,66,67] as well as by Bartlett’s test of sphericity from which significant values would be expected (*p* < 0.001). In addition, the number of factors was determined by parallel analysis and eigenvalues and set in the initially proposed dimensions based on the literature review, and the maximum likelihood factor extraction method and the oblimin rotation method were used.

In turn, two models were tested for the CFA: a first-order model with the purpose of evaluating the factorial validity of the proposed five-dimensional theoretical construct [68] and a second-order model with the purpose of validating the existence of a higher-order factor that generates the first-order factors that in turn generate the observed responses [36,69]. The first model was evaluated with the recommended robust MLM estimation method because it did not meet the multivariate normality assumption [70]. Feasibility of the models was determined by chi-square (χ^2^), comparative fit index (CFI), Tucker–Lewis index (TLI), root mean square error of approximation (RMSEA), and standard root mean square residual (SRMR) [71]. In addition to χ^2^, the calculation of the relative chi-square for the model (χ^2^/df) was included, where values between 2 and 3 indicate a good model fit [72].

Calculations of the magnitude of fit indices, such as SRMR < 0.08, RMSEA < 0.05, and CFI and TLI > 0.90 or 0.95, were included [64,73]. In addition, since it is assumed that the five dimensions measure the same construct, a second-order CFA was performed. Similarly, in the first model, the magnitude of factor loadings (λ) was included, being adequate when they are > 0.70 [74]. Additionally, for evidence of internal validity, through convergent validity, the average variance extracted (AVE) per factor was calculated (AVE > 0.50). Interfactorial correlations (φ) were also calculated according to conceptual affinity, since the evidence of discriminant validity is evaluated by empirical differentiation between the AVE and the square of the interfactorial correlations (φ^2^) where the former is expected to be greater (AVE > φ^2^) [75]. As for the reliability estimation, the alpha coefficient, ω coefficient to assess the construct, and H coefficient (>0.70) were taken into account [76,77].

Descriptive analyses were performed with R codes. The EFA was performed with the psych package [78], together with reliability and inter-factor correlations through the Jamovi 1.6.22 interface, while the CFA was carried out using the Lavaan package in the R-Studio interface.

## 3. Results

### 3.1. Content Validity

The results of the content validity of the 43 items of the Q-COPE are shown in Table 1. For the evaluation of the items, the critical value of V = 0.5 was considered for the identification of the items that should be revised or withdrawn, i.e., if the critical value was within the confidence interval of the item, it would be considered for revision or rejection [60]. Five items included the critical value (marked with an asterisk in Table 1). Since there were few, it was considered appropriate to consider them in the EFA.

### 3.2. Descriptive Statistics of the Q-COPE Items

The descriptive analyses of the Q-COPE are shown in Table 1. The data for the EFA showed that the 43 initial items of the scale presented asymmetry and kurtosis <|2|, so it was decided to perform the EFA with Pearson’s correlation matrix. The data for the CFA also showed univariate skewness and kurtosis within the expected ranges, while for multivariate normality it exceeded the established criteria (Mardia kurtosis = 138.23; *p* < 0.001), so it was decided to use the MLM robust maximum likelihood factor estimation method for the CFA.

### 3.3. Evidence of Validity of the Internal Structure

**Figure 1:**
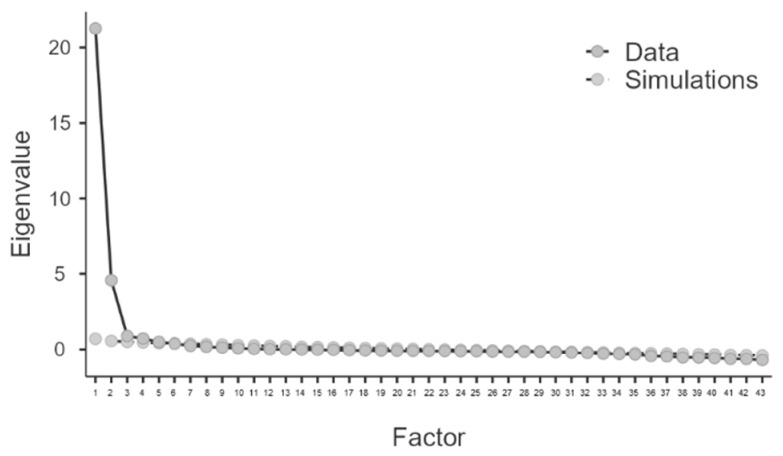
Number of factors based on eigenvalue.

The KMO = 0.976 was sufficiently high and Barlett’s test of sphericity was significant (*p* < 0.001) for the proposed five-factor scale. The parallel analysis indicated that it would be convenient to extract five factors confirming the initial proposal. The sedimentation plot also indicated a five-factor solution (Figure 1).

For EFA, we were subjected to the maximum likelihood extraction method and the oblimin rotation method, iteratively eliminating the items whose loadings were less than 0.40 in the proposed factors (2 to 5, 8, 12 to 14, 16, 20, 23, 28 to 33, 37, 38 and 43), leaving 23 items with factor loadings greater than 0.4, with individual communality > 0.5. Therefore, they were greater than the minimum required (h2 < 0.30) [79,80] and did not alter the results of the factor analysis [81]. Of the five factors, Emotional regulation explained 22.6% of the variance; Information, 18.4%; Social support, 14.4%; Altruism, 12.6%, and Accommodation, 11.6% (Table 2).

The factor structure proposed by the EFA was subjected to CFA (See Supplementary Materials). Therefore, with the second sample, two models were tested, a model with five first-order factors (Table 2) and a second-order model to assess the whole coping quarantine construct (Figure 1). The first model shows adequate fit indices (Table 3): χ^2^ = 489.048, df = 220, χ^2^/df = 2.223, SRMR = 0.025, CFI = 0.969, TLI = 0.965, RMSEA = 0.047 (90% CI = 0.043; 0.051), factor loadings had acceptable magnitudes (λ > 0.70). Similarly, for convergent validity, the AVE reached an acceptable magnitude (>0.50) and are robust for each factor. In terms of internal discriminant validity, it was observed that the AVE are higher than the shared variance between factors (AVE > φ^2^) in all sections, except for the social support and information factor. Likewise, correlations above 0.80 may indicate multicollinearity [82,83]. This would suggest the existence of a higher-order factor, so it was decided to perform a second-order CFA [84,85].

**Figure 2:**
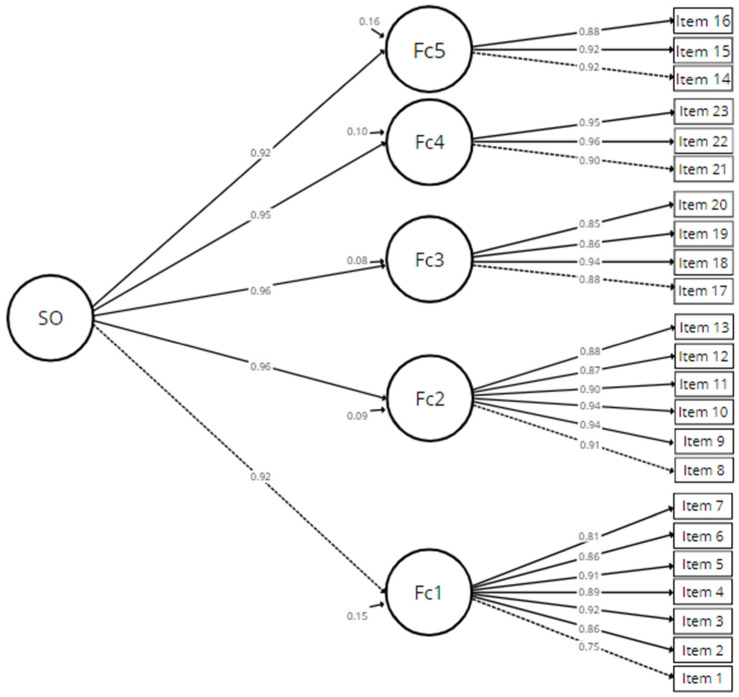
CFA of the second order model. Confirmatory factor analysis of the 5-factor model with a second order factor, SO (Second Order Factor) = Quarantine coping strategies; Fc1 (Factor 1) = Emotional regulation; Fc2 (Factor 2) = Information; Fc3 (Factor 3) = Social support; Fc4 (Factor 4) = Altruism; Fc5 (Factor 5) = Accommodation.

A second model was evaluated (Figure 2) with the purpose of obtaining evidence to interpret the instrument as a multilevel scale in which a higher order factor brings together the five dimensions [36,69]. Performing the CFA suggested adequate fit indices: χ^2^ = 499.674, df = 225, χ^2^/df = 2.221, SRMR = 0.026, CFI = 0.969, TLI = 0.965, RMSEA = 0.047 (90% CI = 0.043; 0.051), some of which showed better fit values than the first-order factor model and others which were equal (Table 3).

### 3.4. Internal Consistency

Internal consistency (Table 2) was evaluated by Cronbach’s Alpha (α), ordinal Alpha (ordinal α), McDonald’s Omega (ω), and H coefficient (α), obtaining higher values than 0.90. Moreover, an α of 0.98 and a ω of 0.98 were obtained for the second order model. In addition, an α of 0.98 and a ω of 0.98 were obtained for the second order model.

## 4. Discussion

A number of studies during pandemics have used various instruments that assess mental health. However, few studies have been conducted during quarantine as a contingency measure for a catastrophic event to evaluate coping strategies.

Taking this into account the ways of thinking and acting of each individual, the Q-COPE was developed using a five-point model based on recent literature that considers factors: [19,38] (a) emotional regulation, which refers to the ways of thinking and behaving that allow the recognition of potentially beneficial aspects of a quarantine, considering it as an opportunity for personal development and family ties, coinciding with the coping strategies reported in the individual experience; [39] (b) information measures the access that the person has to the information necessary to act adequately in the face of a disease that requires mandatory social isolation, being an important factor according to what has been reported in the scientific literature; [14,40] (c) social support refers to the ways of thinking and acting that allow strengthening the security of interacting adequately with sources of support, family and professional, through different means of bidirectional communication, considered an external support supported by previous studies; [41,86] (d) altruism, recognizes that keeping quarantine contributes to the welfare of others, especially those in vulnerable situations; [19] and (e) accommodation, measures the person’s adjustment to the limitations of basic survival supplies in quarantine situations, such as food and personal hygiene items, and is linked to government actions [12,39,45,46]. Therefore, the objective of this research was to develop and analyze the psychometric properties of a scale to evaluate coping strategies in the face of quarantine, in the context of social isolation in a Peruvian population, during the COVID-19 pandemic.

Regarding the evidence of validity of the internal structure, the 5-factor structure initially proposed was corroborated. The EFA sought to explore the proposed structure, which presented an adequate fit considering the proposed theory, given that the items belong to separate dimensions for coping strategies during quarantine. [19]

Although the five factors explain 79.6% of the variance, the correlations between factors are high, which may suggest unidimensionality [87]. Therefore, the interpretation of the 5 dimensions would not be justified. To test the adequacy of the empirical data, a second-order model (quarantine coping as a general factor) was proposed. The first-order structure obtained similar adjustment indexes with respect to the second-order structure, empirically verifying the defined structure, i.e., the results of the second-order CFA provided an opportunity to confirm the five dimensions of the first-order structure. In this sense, the first-order structure of the Q-COPE, suggested in the present work, is akin to the domain-specific conception of quarantine coping, given that the assessment depends on the domain in which it operates. However, the Q-COPE also supports a complete sense of the construct: coping during quarantine encompassing emotional regulation, information, accommodation, social support, and altruism. Furthermore, the results showed clear evidence of concurrent validity. (AVE > 0.50). The discriminant validity (AVE > φ^2^) was satisfactory, except for the items of the social support factor, which failed to discriminate with the items of the information factor. Despite this result, it is not conclusive, as the debate continues about considering discriminant validity as a criterion for evaluating measurement instruments. Although various methods are presented for the evaluation of discriminant validity, this should not be done statistically, since they may lead to erroneous conclusions on the suitability of the scale [88]. On the contrary, discriminant validity should be performed in a theoretical way that comprises a content validity. This has been widely discussed and recommended by Borsboom et al. [89]. Thus, when there is evidence of the existence of several models that can explain the data in a very similar way, these models are considered equivalent because they represent a hypothetical construct in a different way but are statistically identical. For this reason, it is necessary to choose between these models, based on the results, existing theory, or logic to argue in favor of one over the others [88]. In the present study, we consider that by reporting the existence of a second-order factor for other coping models, there is theoretical and logical support based on the results for choosing the second-order model as the one that best explains the findings [89].

Regarding reliability, the indicators were high (>90). In addition, we considered the analyses (ordinalα, ω and H), whose magnitudes were adequate, since these coefficients are considered better estimators versus alpha (α), which tends to underestimate reliability [90]. Although the evidence indicates a favorable internal structure, further studies can be completed by comparing the instrument in other contexts. The findings of this study allow us to calculate the general factor score of the second-order model and the specific factors independently [91,92,93] and it is considered that both the first and second-order models, taken together, provide evidence for a structure for the five first-order factors and one second-order factor. From a theoretical point of view, it is important to highlight that there is no instrument capable of replicating a multidimensional construct that evaluates coping strategies during quarantine. Therefore, the practical implications of the Q-COPE are that it can be applied in contexts that link other variables associated with the construct, such as resilience, hope, optimism, stress coping strategies, positive/negative emotions, anger, anxiety, depression, and other psychological variables of importance in the context of quarantine. In practical terms, this instrument could be implemented as a complement to the psychological evaluations carried out by mental health professionals, with the purpose of verifying the coping strategies of people in a situation of quarantine through cross-sectional or longitudinal studies. It can also be useful as a tool to evaluate the effectiveness of intervention programs aimed at developing coping strategies during quarantine, as well as to verify the psychological effects of procedures that have been implemented in specific situations. Examples of the application of the instrument includes its use to affront a new disease that implies the implementation of a quarantine, to verify the relationship between quarantine coping strategies to affront prolonged duration, fear of infection, financial loss, adequate or inadequate information [7], lack of sources like food or water [18], such as depression [9], anxiety [11], frustration [12], uncertainty, psychological distress in parents [20], other variables like educational level, age, people studying during quarantine, people living alone or with previous physical conditions or those with recent health problems under conditions of social isolation [13,14,15]. Additionally, the relationship between quarantine coping with protective factors can be studied, e.g., resilience adaptive perception of control [22,23]. Other variables of interest may be healthy habits, nutrition styles, sleeping habits, and so on [21].

Although the sample size is adequate considering the items, dimensions, and magnitude of factor loadings [94], some limitations are found, such as high interfactor correlations despite a large sample size, which reinforces the argument that large sample sizes are not indispensable at this point [95]. The sample was non-probabilistic, so it is not possible to extrapolate results. Likewise, diversification and expansion of the sample to other age groups is recommended. Moreover, measurement invariance between sexes or other groups was not tested, so group differences should be analyzed using an invariance approach to explore construct stability using a predictive or longitudinal invariance approach. Therefore, future studies could incorporate invariance analysis. In addition, the study focused on the evaluation of the internal structure of the Q-COPE and did not consider other sources of validity evidence, such as the relationship with other variables. However, despite its limitations, the study provides a starting point for examining psychological health in a quarantine context in Spanish-speaking countries.

## 5. Conclusions

The present study presents initial evidence of a Spanish-language measure of Q-COPE. Despite the limitations, the findings suggest adequate psychometric properties to assess coping strategies to quarantine in the Peruvian population through five factors: emotional, information, social support, altruism, and accommodation. It is recommended that it be extended to other populations to gather more evidence on the nature of the data.

## Figures and Tables

**Table 1:** Content validity and descriptive statistics of the Q-COPE items.

	V [IC 95%]	M1				M2			
Item		M	DE	g1	g2	M	DE	g1	g2
Item1	0.92 [0.74; 0.98]	3.58	1.30	−0.74	−0.56	3.56	1.33	−0.74	−0.62
Item2	0.79 [0.60; 0.91]	3.48	1.16	−0.40	−0.81	3.51	1.20	−0.48	−0.79
Item3 *	0.67 [0.47; 0.82]	3.34	1.18	−0.23	−0.95	3.35	1.19	−0.36	−0.84
Item4 *	0.67 [0.47; 0.82]	3.05	1.26	0.07	−1.14	3.05	1.31	0.09	−1.20
Item5	0.75 [0.55; 0.88]	3.17	1.31	−0.08	−1.20	3.20	1.32	−0.13	−1.25
Item6	0.96 [0.80; 0.99]	3.71	1.27	−0.93	−0.19	3.76	1.28	−1.01	−0.08
Item7	0.96 [0.80; 0.99]	3.87	1.29	−1.13	0.16	3.90	1.27	−1.11	0.15
Item8	0.71 [0.51; 0.85]	3.42	1.16	−0.31	−0.80	3.49	1.18	−0.47	−0.66
Item9	1.00 [0.86; 1.00]	3.72	1.22	−0.89	−0.14	3.76	1.27	−0.98	−0.07
Item10	0.96 [0.80; 0.99]	3.79	1.28	−1.00	−0.04	3.81	1.29	−1.04	−0.01
Item11	0.96 [0.80; 0.99]	3.63	1.26	−0.81	−0.34	3.68	1.29	−0.83	−0.38
Item12	0.75 [0.55; 0.88]	3.69	1.14	−0.66	−0.39	3.72	1.18	−0.67	−0.47
Item13	0.71 [0.51; 0.85]	3.30	1.17	−0.22	−0.84	3.40	1.22	−0.28	−0.91
Item14	0.79 [0.60; 0.91]	3.34	1.18	−0.26	−0.83	3.39	1.23	−0.27	−0.95
Item15	1.00 [0.86; 1.00]	3.50	1.29	−0.65	−0.62	3.53	1.26	−0.73	−0.45
Item16	1.00 [0.86; 1.00]	3.18	1.24	−0.02	−1.13	3.08	1.30	0.03	−1.22
Item17	0.96 [0.80; 0.99]	3.86	1.28	−1.16	0.27	3.89	1.27	−1.12	0.17
Item18	0.92 [0.74; 0.98]	3.87	1.28	−1.17	0.28	3.92	1.29	−1.20	0.31
Item19	0.88 [0.69; 0.96]	3.83	1.27	−1.13	0.24	3.84	1.27	−1.11	0.16
Item20	1.00 [0.86; 1.00]	3.39	1.14	−0.30	−0.81	3.40	1.25	−0.32	−0.98
Item21	1.00 [0.86; 1.00]	3.69	1.24	−0.96	−0.07	3.77	1.26	−1.02	0.02
Item22	0.96 [0.80; 0.99]	3.62	1.24	−0.89	−0.18	3.67	1.25	−0.91	−0.15
Item23	1.00 [0.86; 1.00]	3.54	1.24	−0.78	−0.37	3.60	1.21	−0.73	−0.39
Item24	0.96 [0.80; 0.99]	3.80	1.25	−1.10	0.19	3.81	1.27	−1.07	0.09
Item25	1.00 [0.86; 1.00]	3.57	1.26	−0.81	−0.40	3.65	1.26	−0.85	−0.30
Item26	1.00 [0.86; 1.00]	3.63	1.24	−0.90	−0.20	3.68	1.28	−0.91	−0.27
Item27	1.00 [0.86; 1.00]	3.57	1.25	−0.80	−0.35	3.59	1.26	−0.79	−0.41
Item28 *	0.63 [0.43; 0.79]	2.64	1.15	0.18	−0.82	2.68	1.25	0.26	−0.92
Item29 *	0.63 [0.43; 0.79]	2.75	1.15	0.12	−0.89	2.77	1.22	0.12	−0.97
Item30	0.92 [0.74; 0.98]	3.05	1.24	0.13	−1.04	2.98	1.30	0.12	−1.12
Item31 *	0.54 [0.35; 0.72]	3.49	1.12	−0.39	−0.69	3.42	1.20	−0.33	−0.85
Item32	0.71 [0.51; 0.85]	3.20	1.21	−0.27	−0.91	3.24	1.24	−0.33	−0.89
Item33	0.96 [0.80; 0.99]	3.48	1.26	−0.75	−0.51	3.55	1.22	−0.80	−0.35
Item34	0.96 [0.80; 0.99]	3.63	1.33	−0.76	−0.61	3.72	1.29	−0.88	−0.37
Item35	0.92 [0.74; 0.98]	3.67	1.27	−0.94	−0.17	3.72	1.24	−1.00	0.05
Item36	1.00 [0.86; 1.00]	3.52	1.26	−0.66	−0.66	3.54	1.28	−0.70	−0.59
Item37	1.00 [0.86; 1.00]	3.63	1.29	−0.81	−0.48	3.69	1.23	−0.94	−0.05
Item38	0.96 [0.80; 0.99]	3.59	1.24	−0.88	−0.15	3.65	1.21	−0.88	−0.11
Item39	1.00 [0.86; 1.00]	3.49	1.28	−0.64	−0.69	3.54	1.26	−0.70	−0.54
Item40	1.00 [0.86; 1.00]	3.69	1.29	−0.93	−0.23	3.74	1.25	−0.95	−0.09
Item41	1.00 [0.86; 1.00]	3.84	1.29	−1.06	0.00	3.89	1.29	−1.14	0.17
Item42	1.00 [0.86; 1.00]	3.82	1.31	−1.06	−0.05	3.88	1.28	−1.14	0.22
Item43	0.96 [0.80; 0.99]	2.60	1.30	0.54	−0.85	2.54	1.31	0.58	−0.84

Note. M1 = Sample for EFA, M2 = Sample for ACF, M = Mean, SD = Standard Deviation, g1 = skewness, g2 = kurtosis, V = Aiken’s V, 95% CI = 95% confidence interval, * items with critical value V = 0.5 within confidence interval.

**Table 2:** EFA, CFA, correlation, validity, and reliability of the five-dimensional model.

EFA							CFA					
Initial Numbering	F1	F2	F3	F4	F5	h2	F1 (λ)	F2 (λ)	F3 (λ)	F4 (λ)	F5 (λ)	Final Numbering
1	0.48					0.610	0.75					1
6	0.75					0.728	0.86					2
7	0.84					0.814	0.92					3
9	0.92					0.810	0.89					4
10	0.93					0.842	0.91					5
11	0.85					0.751	0.86					6
15	0.53					0.599	0.81					7
17		0.84				0.869		0.91				8
18		0.89				0.927		0.94				9
19		0.67				0.857		0.94				10
21		0.61				0.807		0.90				11
22		0.56				0.740		0.87				12
24		0.40				0.848		0.88				13
25					0.73	0.802					0.92	14
26					0.64	0.797					0.92	15
27					0.69	0.769					0.88	16
34			0.75			0.756			0.88			17
35			0.73			0.853			0.94			18
36			0.77			0.740			0.86			19
39			0.66			0.726			0.85			20
40				0.63		0.813				0.90		21
41				0.83		0.921				0.96		22
42				0.92		0.932				0.95		23
% variance	22.6	18.4	14.4	12.6	11.6	Complete scale						Complete scale
α	0.95	0.97	0.93	0.96	0.92	0.98	0.95	0.97	0.93	0.95	0.93	0.98
ω	0.95	0.97	0.93	0.96	0.92	0.98	0.95	0.97	0.94	0.95	0.93	0.98
-	-	-	-	-	-	-	0.95	0.97	0.93	0.95	0.93	_Ordinal_α
-	-	-	-	-	-	-	0.96	0.97	0.94	0.96	0.94	H
-	-	-	-	-	-	-	0.74	0.82	0.78	0.88	0.82	AVE
-	-	-	-	-	-	-	0.74	0.82	0.78	0.88	0.82	F1
-	-	-	-	-	-	-	1	0.79	0.76	0.77	0.71	F2
-	-	-	-	-	-	-	0.89	1	0.83	0.81	0.77	F3
-	-	-	-	-	-	-	0.87	0.9	1	0.85	0.79	F4
-	-	-	-	-	-	-	0.87	0.9	0.91	1	0.72	F5

Note. F1 = Emotional regulation; F2 = Information; F3 = Social support; F4 = Altruism; F5 = Accommodation. α: Cronbach’s alpha; _Ordinal_α: Ordinal alpha; ω = McDonald’s Omega; λ = Factor loading; AVE: average variance extracted; below the diagonal: interfactor correlations; above the diagonal: variance shared between factors (AVE > φ^2^).

**Table 3:** Goodness-of-fit indices obtained in the CFA.

Model	χ^2^ (df)	*p*-Valor	χ^2^/df	SRMR	CFI	TLI	RMSEA [90% CI]
First Order	489.048 (220)	0.000	2.223	0.025	0.969	0.965	0.047 [0.043; 0.051]
Second Order	499.674 (225)	0.000	2.221	0.026	0.969	0.965	0.047 [0.043; 0.051]

Note: M—1 = Model with 5 factors; M—2 = Model with 5 factors and a second order factor.

## Data Availability

The data can be requested from the author by correspondence.

## Supplementary Materials

The following supporting information can be downloaded at: https://www.mdpi.com/article/10.3390/ijerph192214847/s1, Table S1: Escala de Afrontamiento ante una cuarentena (EAC).
