# Supplementary material for: Psychometric Analysis of the Quarantine Coping Strategies Scale (Q-COPE) in the Spanish Language
Source: Int J Environ Res Public Health. 2022 Nov 11;19(22):14847. doi: 10.3390/ijerph192214847 (PMC9690774; doi:10.3390/ijerph192214847)
Supplement: Supplementary file 1 [file ijerph-19-14847-s001.zip › ijerph-1955008-supplementary.pdf]

Table S1. Escala de Afrontamiento ante una cuarentena (EAC).

		TED	ED	NAND	DA	TED
1	Considero que tengo la capacidad emocional para afrontar todo el tiempo que dura una cuarentena					
2	Considero que el tiempo de cuarentena puede ser aprovechado para desarrollar aspectos de mi vida que en otras circunstancias sería más difícil mejorar					
3	El tiempo de cuarentena pasado en familia puede fortalecer los vínculos familiares					
4	Veo el tiempo que dura una cuarentena como una excelente oportunidad para desarrollar capacidades y habilidades nuevas					
5	El tiempo que dura una cuarentena me permite mejorar mis vínculos familiares					
6	El tiempo de duración de una cuarentena puede ser muy útil para aprender a encontrar momentos de paz y calma					
7	Estoy seguro que al finalizar una cuarentena seré una mejor persona de lo que era antes					
8	Estoy informado acerca de los hábitos de higiene que debo tener para evitar o reducir la propagación de una enfermedad durante una cuarentena					
9	Me he informado del uso correcto de accesorios (mascarillas, guantes, etc.) que generalmente se usan para evitar o reducir la propagación de una enfermedad que obliga a establecer una cuarentena					
10	Estoy plenamente informado sobre las medidas sanitarias generales que debemos adoptar las personas durante una cuarentena					
11	Me siento tranquilo porque sé cómo informarme para evitar o disminuir el riesgo de propagación de una enfermedad que requiere la realización de una cuarentena					
12	Sé que puedo acceder a la información provista por las autoridades sanitarias que se encuentran a cargo de mantener las epidemias y pandemias bajo control					
13	Comprendo bien las razones generales por las cuales se debe acatar una cuarentena					
14	Es posible para mí tener los alimentos necesarios para pasar por una cuarentena					
15	Mis suministros incluyen material de aseo personal suficiente para enfrentar una cuarentena					
16	En caso de acabarse mis reservas de alimentos, puedo volver a abastecerme en el mínimo tiempo posible					
17	Dispongo de un teléfono móvil con cargador funcionando y con suficiente saldo					
18	Estoy convencido(a) de tener las suficientes habilidades para mantener la comunicación a distancia con mis amigos(as) y familiares durante una cuarentena					
19	Tengo conocimiento de las líneas telefónicas y redes sociales de ayuda establecidas por el gobierno nacional y local durante periodos de cuarentena					
20	Puedo acceder a líneas telefónicas y redes sociales para comunicarme directamente con personal de salud preparado para atender mis necesidades durante una cuarentena					
21	Una de las sensaciones más agradables de la cuarentena es saber que estando en ella se está contribuyendo al cuidado y protección de otras personas					
22	Estoy convencido(a) que al acatar la cuarentena protejo del contagio a las personas más vulnerables: niños, ancianos o con condiciones médicas preexistentes					
23	Estoy convencido(a) que al acatar la cuarentena doy un gran apoyo al trabajo de las autoridades de salud					

Escala de Afrontamiento ante una cuarentena (EAC). A continuación, encontrarás una lista de afirmaciones que tienen que ver con el afrontamiento auto percibido ante una cuarentena, señale usted su respuesta marcando con una X uno de los casilleros que se ubica en la columna derecha,

utilizando los siguientes criterios: **TED** = Totalmente en desacuerdo, **ED** = En desacuerdo, **NAND** = Ni de acuerdo ni en desacuerdo, **DA**= De acuerdo, **TDA** = Totalmente de acuerdo. Recuerda que: tu sinceridad es muy importante, no hay respuestas buenas ni malas, asegúrate de contestar todas.
